# Immunomodulatory Effects of Phosphorylated *Radix Cyathulae officinalis* Polysaccharides in Immunosuppressed Mice

**DOI:** 10.3390/molecules24224150

**Published:** 2019-11-15

**Authors:** Haibo Feng, Jing Fan, Lang Lin, Yunjie Liu, Dongkun Chai, Jie Yang

**Affiliations:** 1 College of Life Science and Technology, Southwest Minzu University, Chengdu, Sichuan 6100041, China; 2 Sichuan Industrial Institute of Antibiotics, Chengdu University, Chengdu, Sichuan 610051, China; fanjingcdu@sina.com; 3 Department of Veterinary Medicine, Southwest University, Rongchang, Chongqing 402460, China; umbrella_fj@163.com (L.L.); lyj_lyj1981@sina.com (Y.L.); fengabcd123@outlook.com (D.C.); yangyanswu@yahoo.com (J.Y.)

**Keywords:** *Radix cyathulaofficinalis kuan* polysaccharide, phosphorylation modification, polysaccharide, immunosuppressed mice

## Abstract

This research aimed to investigate the immunomodulatory effects of phosphorylated *Radix Cyathulae officinalis Kuan* polysaccharides (pRCPS) in immunosuppressed mice, improving their cellular and humoral immune function. Our results showed that pRCPS increased serum immunoglobulin (IgG, IgA, IgM) concentrations significantly, enhanced splenocyte proliferation, and the thymus and spleen indices. pRCPS also promoted phagocytosis in peritoneal macrophages and enhanced cytokine (IFN-γ, IL-2, -4, -5, -6, and -10) serum levels. Importantly, pRCPS increased the proportions of selected T cell subpopulations (CD3^+^, CD4^+^, and the CD4^+^ to CD8^+^ ratio). Our results revealed that phosphorylation of the polysaccharides promoted their immune-enhancing effects. Thus, pRCPS can enhance cellular and humoral immunity and could be used as an immune-enhancing agent to overcome cyclophosphamide (CY)-induced immunosuppression.

## 1. Introduction

Immunosuppression is a common phenomenon in animals and humans and can be caused by disease, trauma, stress, toxins, therapeutic drugs, or medical procedures such as anesthesia. In addition, stress, nutrition, and exercise also have important modulatory effects on the immune system [1]. For example, physiological stress in large animals, diseases like shipping fever in cattle, or early weaning of piglets can cause immunosuppression [2]. In recent years, it has been shown that many infectious diseases can damage the immune system and contribute to immunosuppression in animals. A viral disease known as PRRS (porcine reproductive and respiratory syndrome) has a worldwide distribution which can be economically burdensome to the swine industry [3,4]. When the PRRS virus infects piglets, it invades lung macrophages and impacts adaptive immune responses, leading to delayed generation of IFN-γ secreting cells, reduced immunity, and infection of multiple organs, including interstitial pneumonia, myocarditis, and others. The immune inhibitory effect of this virus can trigger infections by a number of secondary pathogens and may result in a mixed infection [3,4,5,6]. Thus, developing novel immune enhancers to improve immune function in immunosuppressed animals has become a crucial objective in the fields of immunology and pharmacology.

The modulation of the immunity has a key part in the prevention of infectious diseases. Recently, many researchers have focused on the immunomodulatory and immunostimulatory effects of natural products. Multiple studies have shown that polysaccharides extracted from plants can enhance the immune response in animal models of immunosuppression [7,8]. For instance, Li, et al. (2015) reported that the pollen polysaccharide from Taishan *Pinus massoniana* (TPPPS) showed immunomodulatory effects in chickens infected with two immunosuppressive viruses (Avian leukosis and Reticuloendotheliosis viruses) [7]. They showed that TPPPS noticeably improved the levels of specific antibodies, CD4^+^ and CD8^+^ cells, and IFN-γ and IL-2 cytokines in the peripheral blood of immunosuppressed chickens. Wang et al. (2012) investigated the polysaccharide from *Cordyceps militaris* (CMP) and its immune-enhancing effects of in vivo in a cyclophosphamide-induced murine immunosuppression model. Their results showed the role of CMP in increasing the indices of spleen and thymus, the splenocytes’ proliferation and the phagocytic index of macrophages, indicating the role of CMP in upregulating the immunity of CY- immunosuppressed mice [8]. Huang et al. (2016) demonstrated that administering litchi pulp polysaccharides (LP) to cyclophosphamide (Cy)-immunosuppressed mice resulted in enhanced mesenteric lymph node cell proliferation, production of cytokine (TNF-α and IL-6), IgA, IgG, and IgM serum levels, and increased thymus and spleen indices. These results showed that LP triggers immunity of intestinal mucosa, thus leading to immunomodulatory effects [9]. 

Recently, studies have also shown that immunomodulators can augment host defense responses, thus effectively increasing disease resistance [10]. Clinical investigations have proven that boosting immunity can effectively provide disease resistance. Immunotherapy has been proposed for more than a century, and marvelous progress has been achieved in recent years, especially in the field of tumor immunotherapy [11].

A perennial herbaceous plant, *Radix Cyathulae officinalis Kuan* (RC) grows extensively in Asian subtropics. The Chinese traditional medicine widely uses roots of RC to enhance immunity in humans and animals [12,13]. In our earlier study, we extracted *Radix Cyathulae officinalis Kuan* polysaccharides (RCPS) as a water decoction followed by ethanol precipitation. The results showed that they significantly increased both specific and non-specific immunity [12,13]. We also introduced a sulfated modification to RCPS (sRCPS) and investigated its role in the specific humoral and cellular immunity in mice towards a hepatitis B subunit vaccine. The results showed that sRCPS effectively enhanced both humoral and cellular immunity by suppressing the frequency of Treg cells and promoting DC maturation [14]. However, not much is known regarding the pRCPS effects in immunosuppressed animals. 

In this study, we carried out RCPS extraction and purification from RC following the previously described methods, and then phosphorylated the polysaccharides according to a previously reported method. Preliminary structure of pRCPS was characterized by analyzing its physicochemical properties and by infrared spectroscopy (IR). In addition, the immunomodulatory activity of pRCPS was investigated in a Cy-induced immunosuppressive mouse model, and whether the phosphorylation modification in RCPS can improve its immunomodulatory function. 

## 2. Results and Discussion

### 2.1. pRCPS and the PM Phagocytic Function

**Figure 1:**
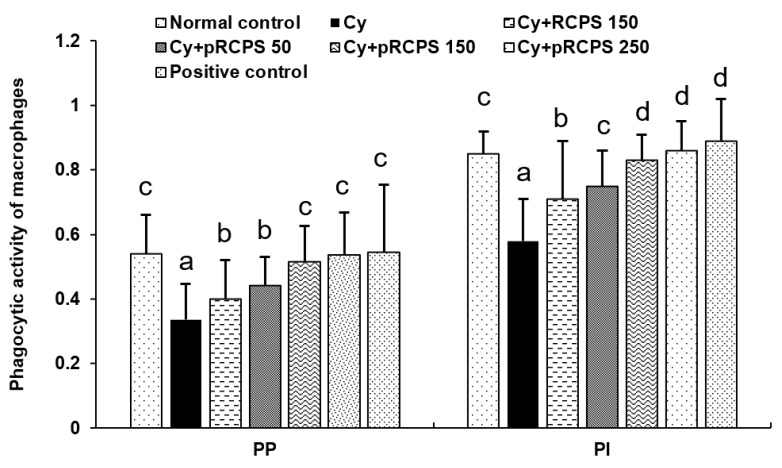
Effects of pRCPS on peritoneal macrophages (PM) in Cy-immunosuppressed mice. After 24 h of the last treatment, 1.0 mL of 3% sterilized starch solution was intraperitoneally injected into the mice. After an additional 24 h, 1.0 mL chicken red blood cells solution (1%) were intraperitoneally injected into the mice. 30 min later, one drop of peritoneal fluid was observed under an optical microscope after placing it on a microscope slide and stained with Wright’s stain. The peritoneal macrophages percent (PP) and peritoneal macrophages percent (PI) were determined. Results are presented as the mean ±SD (*n* = 10). The different letters (a–d) on a column differ significantly (*p* < 0.05).

Macrophages are found in almost all tissues of the body. These cells play a key part in inflammatory and immune responses during pathogenic infections and also contribute to the tissue homeostasis maintenance. One of the most exhaustively-studied macrophage populations include the peritoneal macrophages [15,16]. The phagocytic activity of macrophages is regulated by a lymphocyte factor named Macrophage migration inhibitory factor (MIF) which can enhance phagocytosis and target cell killing. The increase in phagocytic activity is required for resisting pathogenic infections [17]. In the current study, we evaluated the pRCPS effects on the phagocytic role of PM in immunosuppressed mice, and our findings are shown in Figure 1. In comparison to the control group, the PP and PI values were reduced significantly in the Cy immunosuppressed group. Importantly, the PI and PP values increased significantly in the three Cy+pRCPS groups and in the Cy+RCPS group in comparison to the Cy group. Interestingly, the PP and PI values for the pRCPS group were higher than those for the RCPS group. The present results indicate that RCPS and pRCPS significantly increased PM phagocytic function in immunosuppressed mice. The improvement in phagocytic function induced by pRCPS was higher than that induced by RCPS, demonstrating that the phosphorylation modification enhanced the biologic activity of RCPS in immunosuppressed mice.

### 2.2. pRCPS Affects Body Weight and Thymus and Spleen Indices

**Figure 2:**
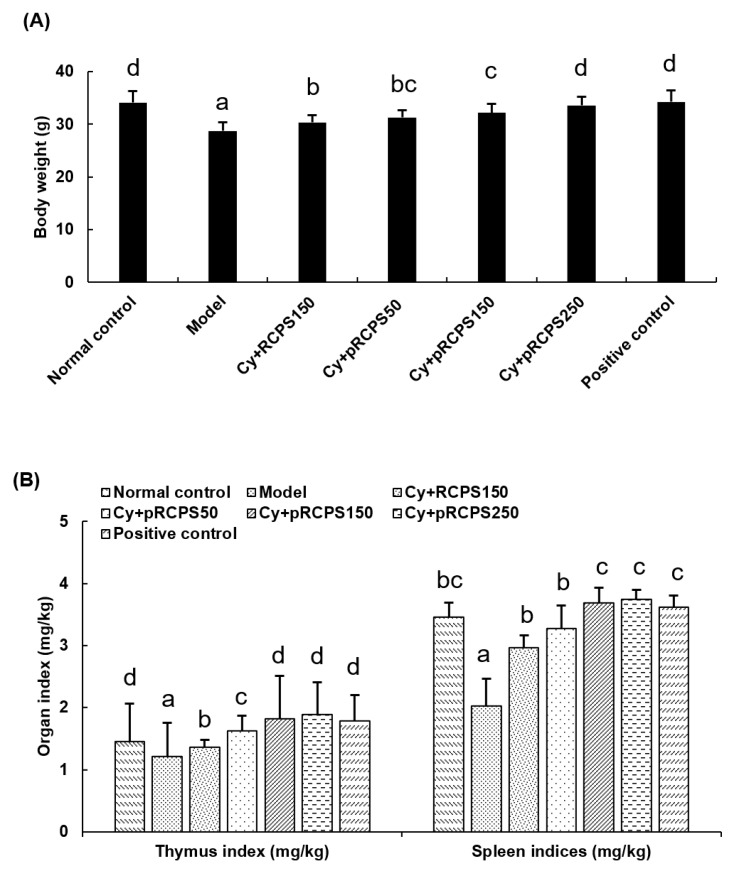
Effects of pRCPS on the Cy-immunosuppressed mice in terms of body weight, spleen, and thymus indices. After 24 h of the last treatment, the weight of body, spleen, and thymus were collected and the (**A**) body weight, (**B**) spleen indices, and thymus indices were determined and calculated. Results are presented as the mean ±SD (*n* = 10). The different letters (a–d) on a column differ significantly (*p* < 0.05).

The status of immunity of an animal are reflected in the indices of lymphoid organs [18]. Some natural polysaccharides can prevent damage to lymphoid organs in immunosuppressed mice. As an example, Li, et al. (2013) investigated *Mosla chinensis Maxim* cv. *jiangxiangru* polysaccharide (MP) in cyclophosphamide-immunosuppressed mice. Their results demonstrated that MP significantly increased the thymus and spleen indices and prevented immune organ damage in mice with suppressed immune systems [18]. Moreover, we also analyzed the body weight in the present study (Figure 2). The body weight and the indices for thymus and spleen in the Cy group were reduced significantly in comparison to the control group (*p <* 0.05). Importantly, the body weight and thymus and spleen indices were higher in the three pRCPS + Cy groups and in the RCPS+Cy group than in the Cy group (*p <* 0.05), indicating that pRCPS significantly augmented the thymus and spleen indices and diminished immune organ damage in immunosuppressed mice. 

### 2.3. pRCPS and Proliferation of Splenocyte

**Figure 3:**
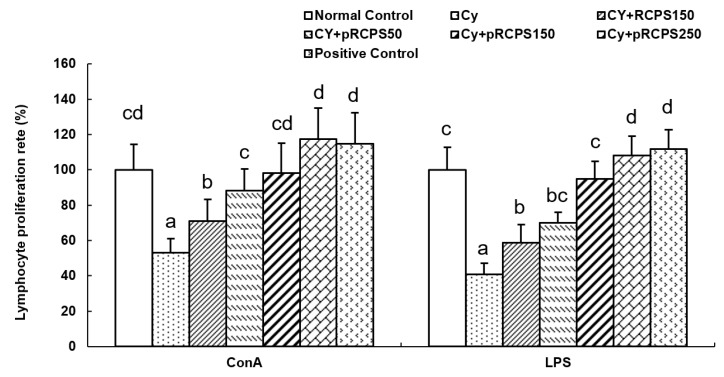
pRCPS affects the proliferation of lymphocyte in Cy-immunosuppressed mice. Cy-immunosuppressed mice were orally treated with pRCPS solution (equivalent to 50, 150, and 250 mg of pRCPS/mouse/day) for 18 days. 24 h after the last dose, splenocytes were extracted and cultured with ConA (5 µg/mL), or RPMI-1640 (control) for 68 h. The cytokine (IL-4 and IFN-γ) concentration of supernatants of splenocyte was measured by ELISA. Data represent the mean ± SD (*n* = 7). Bars marked with different letters (a–d) indicate differences that are statistically significant (*p* < 0.05).

We evaluated the effects of pRCPS on splenocyte proliferative responses by means of the WST-8 method. ConA and LPS were used as mitogens in this experiment. ConA is used to induce T lymphocyte proliferation, whereas LPS can activate B cells and induce B lymphocyte proliferation [19]. Polysaccharides have been previously found to induce similar results in this assay. For example, Zhu et al. (2007) isolated *Ganoderma lucidum* polysaccharides (Gl-PS) and investigated their immune modulatory effects in cyclophosphamide-immunosuppressed mice. Their findings showed that Gl-PS significantly upregulated B and T cell proliferation on day 8 in Cy-treated mice [20]. In the present study, the proliferation of splenocytes is shown in Figure 3. In the Cy group, the lymphocyte proliferation rate was reduced significantly (*p* < 0.05). However, in comparison to the Cy group, the splenocyte proliferative responses of mice treated with RCPS or pRCPS (50,150, 250 mg) were significantly higher (*p* < 0.05). Importantly, the lymphocyte proliferation rates of mice treated with pRCPS (150, 250 mg) were significantly higher than those of mice treated with RCPS. pRCPS significantly enhanced ConA- or LPS-induced lymphocyte proliferation depending on the dose. These outcomes show that pRCPS can counteract the immunosuppressive effects of Cy.

### 2.4. Effects of pRCPS on the Proportion of Peripheral Blood T-Lymphocyte Subsets in Immunosuppressed Mice 

**Figure 4:**
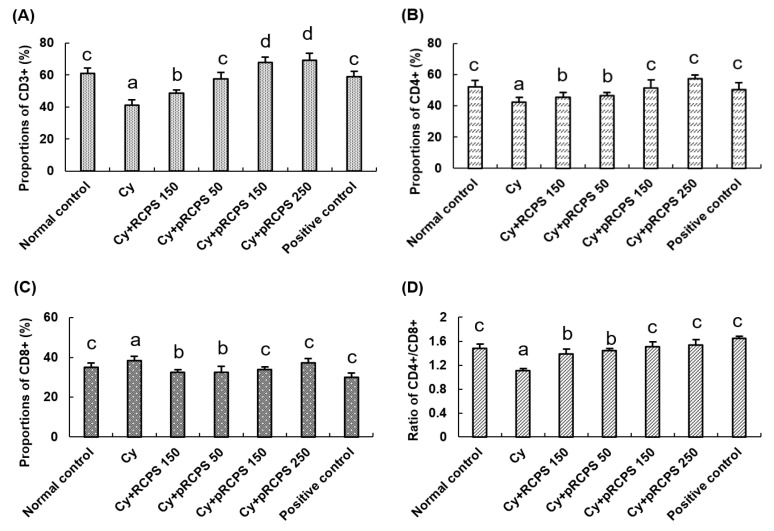
Effects of pRCPS on peripheral blood CD3+, CD4+, CD8+ cells, and on the ratio of CD4+ to CD8+ in Cy- immunosuppressed mice. After 24 h of the last treatment, blood samples were collected and the (**A**) CD3+, (**B**) CD4+, (**C**) CD8+ cells, and (**D**) CD4+/CD8+ were determined by FACS. Results are presented as the mean ± SD (*n* = 10). The different letters (a–d) on a column differ significantly (*p* < 0.05).

T lymphocyte subpopulations play a crucial role in immune responses [21,22,23]. The CD3^+^ antigen is a biomarker that is expressed on the surface of all mature T lymphocytes. T lymphocytes were grouped into two: T inhibitor lymphocytes and T helper lymphocytes. The CD4^+^ antigen is a T helper lymphocytes marker, which assist B cells to secrete antibodies. The CD8+ antigen is a surface marker of T lymphocytes, which can suppress humoral immune responses. When humoral immunity is enhanced, CD4^+^ cells increase while CD8^+^ cells decrease, and the CD4^+^ to CD8^+^ ratio increases. The ratio of CD4^+^ to CD8^+^ thus reviews the activating/inhibitory activity of the immune system [24,25,26]. In the present study, the proportion of Peripheral Blood T-Lymphocyte Subsets is shown in Figure 4. The measure of CD3^+^ and CD4^+^ T lymphocytes and the CD4^+^ to CD8^+^ ratio was reduced significantly in the group treated with Cy in comparison to the normal control group. In contrast, the measure of CD3^+^ and CD4^+^ T lymphocytes and the ratio of CD4^+^ to CD8^+^ in the three pRCPS + Cy groups and in the RCPS + Cy group were significantly higher than in the Cy group. Contrarily, the CD8+ T lymphocytes measure in the three pRCPS + Cy groups was significantly lower than in the Cy and RCPS groups. These results indicate that both pRCPS and RCPS can enhance immune function partly by increasing selected T cell subpopulations in immunosuppressed mice, with pRCPS showing a stronger effect than RCPS. They also suggest that the phosphorylation modification can potentiate the biological effects of RCPS on T cell subpopulations. Along similar lines, Yan et al. (2014) reported that polysaccharides isolated from *Flammulina velutipes mycorrhizae* (PFVM) could increase the measure of CD3^+^ and CD4^+^ T lymphocytes, as well as the ratio of CD4^+^ to CD8^+^ [22]. Shang et al. (2014) investigated the immunomodulatory role of polysaccharides extracted from *Gynostemma pentaphyllum* Makino (GPMPP) in mice that were Cy-immunosuppressed and found that GPMPP increased the measure of CD4^+^ T lymphocytes and the CD4^+^ to CD8^+^ ratio [27].

### 2.5. pRCPS Effects on Cytokine Serum Concentrations in Immunosuppressed Mice

**Figure 5:**
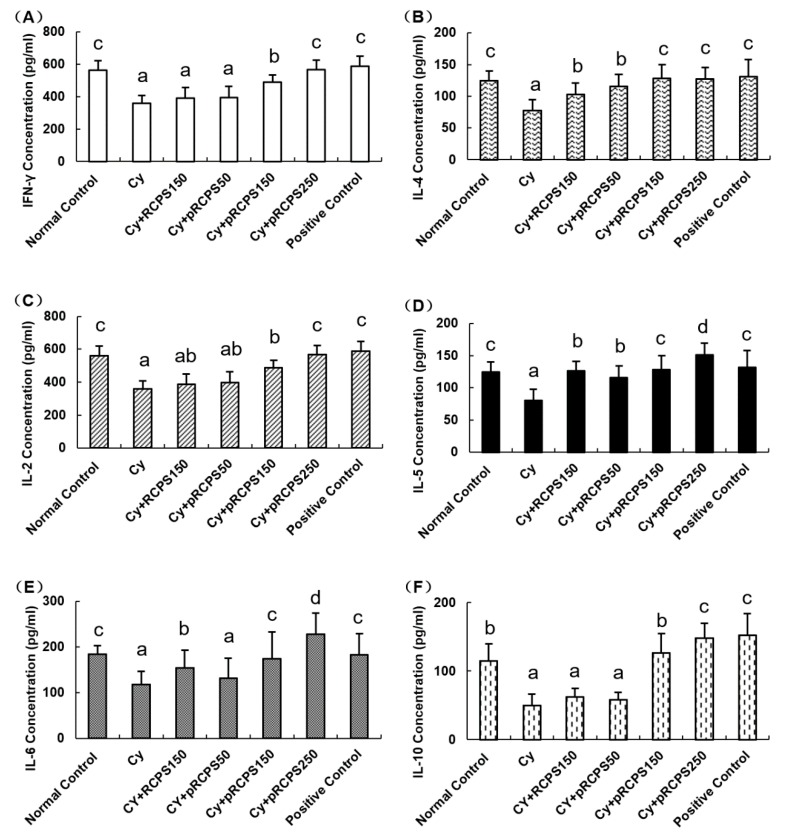
pRCPS effect on serum cytokines in immunosuppressed mice. After 24 h of the last treatment, serum samples were collected and the (**A**) IFN-γ, (**B**) IL-4, (**C**) IL-2, (**D**) IL-5, (**E**) IL-6, and (**F**) IL-10 were determined by ELISA. Results are presented as the mean ±SD (*n* = 10). Bars marked with different letters (a–d) indicate differences that are statistically significant (*p* < 0.05).

Cytokines are important proteins which can modulate the functions of the T helper cellular. [28]. In general, Th1-cytokines enhance division and proliferation of lymphocytes, facilitate maturation of DC, and activate Th1-based immune responses [29]. Th2 cytokines, such as IL-4, -5, -6, and -10, promote Th2-type immune responses and increase antibody production [30,31]. Our results showed that the IL-2, -4, -5, -6, -10, and IFN-γ concentrations in the Cy group were appreciably lower compared to the control group. Compared with the Cy and Cy + RCPS groups, the cytokine (IL-2, -4, -5, -6, -10, and IFN-γ) concentrations in the Cy+pRCPS groups (150, 250 mg) and in the positive control group were enhanced significantly (*p* < 0.05; Figure 5). Also, compared with the Cy group, the IL-4 and IL-5 concentrations in the Cy + RCPS and Cy + pRCPS (50 mg) groups were significantly increased (*p* < 0.05; Figure 5B,D). Thus, pRCPS significantly enhanced Th1 and Th2 cytokines production in Cy-immunosuppressed mice. The enhancement of cytokine production with pRCPS was stronger than with RCPS, indicating that the phosphorylation modification significantly potentiated the immunological activity of RCPS in immunosuppressed mice.

### 2.6. Effects of pRCPS on Serum IgM, IgA, and IgG Concentrations in Immunosuppressed Mice

**Figure 6:**
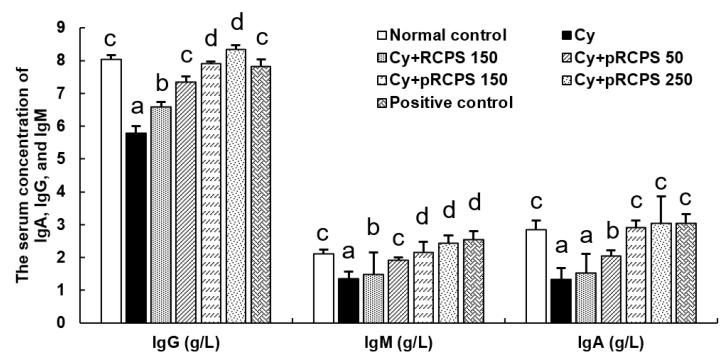
Effects of pRCPS on IgA, IgG, and IgM serum concentrations in Cy-immunosuppressed mice. After 24 h of the last treatment, serum samples were collected and the IgA, IgG, and IgM were determined by ELISA. Results are presented as the mean ±SD (*n* = 10). Bars marked with different letters indicate differences that are statistically significant (*p* < 0.05).

Serum immunoglobulins (Igs) are crucial biomarkers of humoral immunity. IgG, IgM, and IgA are three classes of Ig which jointly represent almost the total amount of serum Ig [32,33]. To assess the pRCPS effects on humoral immunity in immunosuppressed mice, the concentrations of IgA, IgG, and IgM in sera were measured by ELISA. As illustrated in Figure 6**,** the IgG, IgM, and IgA levels in the Cy group were significantly less than in the control group, indicating that Cy suppressed humoral immune function (*p* < 0.05). In contrast, the IgG, IgA, and IgM concentrations in the Cy + pRCPS (50, 150, 250 mg) groups were increased significantly (*p* < 0.05). Thus, pRCPS can improve humoral immunity in Cy- immunosuppressed mice. Huang et al. (2016) investigated the immunomodulatory effects of LP in Cy-immunosuppressed mice and found that administering LP (50, 100, and 200 mg/kg/d) significantly increased IgM, IgA, and IgG levels in the mice sera [9]. 

### 2.7. pRCPS Effects on Cytokine Secretion of Splenocytes in Immunosuppressed Mice

**Figure 7:**
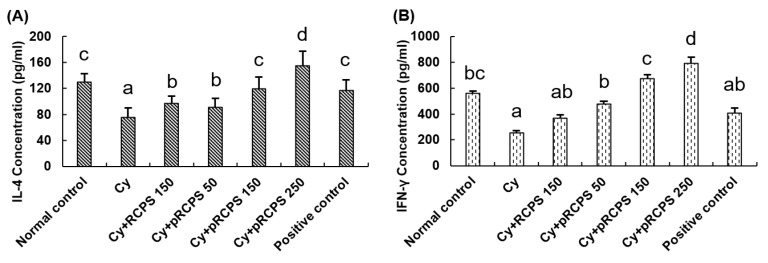
The effects of pRCPS on IL-4 (**A**) and IFN-γ (**B**) level in the supernatants of splenocytes in Cy-immunosuppressed mice. Cy-immunosuppressed mice were orally treated with pRCPS solution (equivalent to 50, 150, and 250 mg of pRCPS/mouse/day) for 18 days. 24 h after the last dose, splenocytes were isolated and cultured with ConA (5 µg/mL) or RPMI-1640 (control) for 48 h. The cytokine (IL-4 and IFN-γ) concentration of supernatants of splenocyte was measured by ELISA. Data represent the mean ±SD (*n* = 7). Bars marked with different letters indicate differences that are statistically significant (*p* < 0.05).

The cytokine secretion activity of splenocytes is a crucial biomarker of immune function of animal [12,34]. Many researchers reported that natural polysaccharides can increase the cytokine production activities of splenocytes in vivo [12,34,35]. In the present study, the effects of pRCPS on the production of cytokines IFN-γ and IL-4 from ConA-stimulated splenocytes in immunosuppressed mice were investigated by ELISA. As illustrated in Figure 7, the concentration of IFN-γ and IL-4 was significantly decreased in Cy group as compared with normal control group (*p* < 0.05). RCPS and three dose pRCPS dramatically enhanced IFN-γ and IL-4 production by splenocytes in Cy induced immunosuppressed mice (*p* < 0.05). It revealed that RCPS and pRCPS significantly enhanced the production of the Th1 and Th2 cytokines in the immunosuppressed mice.

## 3. Experimental Section

### 3.1. Kits and Reagents 

Cyclophosphamide (CY) and Cell Counting kit-8 were procured from the Beyotime Institute of Biotechnology (Jiangsu, China). Propidium iodide, ConA (concanavalin A), media (RPMI-1640), and ELISA kits for mouse cytokines (IL-2, -4, -5, -6, -10, and IFN-γ) were purchased from Wuhan Boster Biological Technology, (Hubei, China). l-glutamine, benzylpenicillin and streptomycin (both 100 IU/ mL), streptomycin (100 μg/mL), fetal bovine serum, and penicillin (100 U/mL) were procured from Gibco Chemical Co. (Saint Louis, Missouri, USA). Anti-mouse anti-CD8-PE, anti-CD4-PE, and anti-CD3-PE monoclonal antibodies that were fluorescent-labeled were obtained from eBiosciences (San Diego, CA, USA). ELISA kits for IgG, IgA, and IgM were purchased from Solarbio Biological Products Co. Ltd. (Chengdu, China).

### 3.2. RCPS and pRCPS Preparation

RCPS were extracted in our lab as previously mentioned [13,36]. Briefly, the dry powder of RC was extracted in water three times under reflux. The aqueous extract was filtered, centrifuged for 10 min at 6000× *g*, and the supernatant were precipitated with ethanol (95%) for 12 h at 4 °C. The protein of precipitate was removed by the Sevag method, dialyzed in a dialysis sack against distilled water for 48 h, and then precipitated with 95% ethanol at 4 °C for 12 h. The resulting precipitate was centrifuged at 6000× *g* for 10 min, washed three times with ethanol (85%), and then the precipitate was dried and collected. Finally, the precipitate was permeated through ADS-7 macroporous adsorption resin to eliminate other pigments, followed by elution through a column (DEAE Sephadex™ A-25) to separate it from the other carbohydrates. The collected eluates were concentrated and lyophilized to harvest the total RCPS fraction. The carbohydrate content (*w/w*) of RCPS was 96.6%, as determined by the phenol-sulfuric acid method. 

RCPS was phosphorylated according the Zhang’s method [37] with a minor modification. Briefly, Sodium trimetaphosphate and sodium tripolyphosphate complexes in 4: 1 (*w/w*) ratios were prepared, and RCPS was added to the complex at 0.01 g/mL, pH 9 and incubated for seven h at 85 °C with constant stirring. The reaction solution was cooled to room temperature, and the ethanol (95%) in 1:4 (*v/v*) ratio was added and precipitated for 24 h at 4 °C, and then lyophilized. Subsequently, the reaction product was re-dissolved by water, and dialyzed by use a dialysis sack (1 cut-off molecular weight 4000) against distilled water until conductivity dropped from 1 × 104 μs/cm to 160 μs/cm. Each preparation was re-lyophilized to produce the pRCPS. The determination of physicochemical properties, amount of phosphate bound, and infrared spectroscopy analysis were described in our previous studies [36].

### 3.3. Experimental Animals and Design

Mice from Institute of Cancer Research (ICR) (Grade II; females, age: five weeks, weight: 18–22 g) were provided by Sichuan Laboratory Animal Center, (Chengdu, China). 60 mice were divided arbitrarily into six groups (n = 10). All animal procedures were performed as per internationally accepted principles, mentioned in the government of China issued Guidelines for Keeping Experimental Animals and approved by the IACUC, Southwest University, (approval number: 1103241911025764). The control group of mice did not receive Cy. From days 1–3, the other five groups of mice were injected intraperitoneally with Cy at 80 mg/kg/d to induce immunosuppression. One group of the Cy-treated mice was used as a control. From days 4–18, compounds were administered to the mice as listed in Table 1. Cy (0.2 mL) was injected intraperitoneally in mice. The polysaccharides were administered by oral gavage in a 0.2 mL solution. After 24 h of the final administration of pRCPS, all mice were weighted and sacrificed, and the spleen and thymus were removed and weighed immediately. Their indices were then calculated as per the following equation: Organ index (mg/g) = (weight of spleen or thymus / body weight). The tissues and sera were then kept at −80 ℃ until further examinations. A summary of animal groups and treatments are mentioned in Table 1. 

### 3.4. Assay for Splenocyte Proliferation 

The spleens were removed after sacrificing the mice 24 h after the last treatment. Single cell splenocyte suspensions were prepared as previously reported [38]. Briefly, spleens were crushed by pressing them with a syringe plunger’s flat surface against a stainless-steel sieve (200 mesh). After lysis of the red blood cells, the splenocytes were washed twice with medium (RPMI-1640). The number of cells were determined with a hemocytometer by applying the Trypan blue dye exclusion method. When the cells exceeded 95% viability, splenocyte blend at 2.5 × 10^6^ cell/mL were seeded onto 96-well plates at 100 μL/well and stimulated for 68 h with 5 μg/mL Con A or LPS (10 μg/mL). The plates were kept at 37 °C in an atmosphere with humidity and 5% CO_2_ for 68 h. To each well 5 mg/mL of WST-8 solution was deposited at 20 μL/well and plates were kept for additional 4 h. The optical densities at 450 and 570 nm were measured on a microplate reader (Synergy HT, Bio-TEK, FL, USA). The lymphocyte proliferation level was calculated as per the following formula: lymphocyte proliferation rate = test OD value /normal control OD value × 100%. 

### 3.5. Phagocytosis Assay

The activity of Peritoneal macrophages was detected as described previously [39]. Briefly, 24 h after the last treatment, 1.0 mL of 3% sterilized starch solution was intraperitoneally injected into the mice. After an additional 24 h, 1.0 mL chicken red blood cells solution (1%) were intraperitoneally injected into the mice. 30 minutes later, one drop of peritoneal fluid was observed under an optical microscope after placing it on a microscope slide and stained with Wright’s stain. The results are presented as the of phagocytic cells percent (PP) and the phagocytic index (PI). The PP is explained as the peritoneal macrophages percent (PM) that ingested one or more chicken red blood cells. The PI is explained as the average number of chicken red blood cells per PM and was determined as the total number of phagocytized chicken red blood cells divided by the total PM (200 cells). PI = the total number of phagocytized chicken red blood cells/200 macrophages×100%; PP = the number of macrophages phagocytizing one or more chicken red blood cells/200 macrophages ×100%.

### 3.6. Analysis of the Concentration of Serum Cytokines

Blood samples were collected 24 h after the last pRCPS administration and clotting was allowed for 2 h. Then, the isolated serum was kept at −20 °C until use. From this, the cytokine (IL-2, -4, -5, -6, -10, and IFN-γ) concentrations were estimated using the ELISA kits, as per instructions.

### 3.7. Serum Ig concentrations 

The preparation of serum was done as described above. Using the ELISA kits, serum IgA, -G, and -M concentrations were assayed as per manufacturer’s instructions.

### 3.8. T-lymphocyte Phenotyping by Flow Cytometry

Peripheral blood samples or suspensions of single cell splenocyte were prepared according to previously described methods [10,40]. Briefly, 1 × 10^6^ cells/mL of splenocyte suspensions were stained with either anti-CD4-PE or anti-CD3-FITC antibodies (10 μL) at 4 ℃ for 1 h. Following two PBS washes, the cells were fixed using 1% PFA (paraformaldehyde) and assessed with a FACS Calibur from BD Biosciences (USA). The proportion of CD3^+^, -4^+^, and -8^+^ T lymphocytes in the mice peripheral blood were analyzed using the software Cell Quest Pro (BD Biosciences, Franklin Lake, NJ, USA). The results are presented as the percent of CD4+ and CD8+ cells. 

### 3.9. IL-4 and IFN-γ Production by Splenocytes In Vitro 

The spleens were collected in sterile environment and the single splenocyte suspension was prepared 24 h after the last treatment of mice, and the splenocyte (2.5 × 10^6^ cells/mL) were cultured with ConA (5 µg/mL) or RPMI-1640 (control) for 48 h in 96-well culture plates at 37 °C with 5% CO_2_. After 48 h, the culture supernatant was collected for the detection of cytokine (IL-4 and IFN-γ) levels using commercial ELISA kits according to the manufacturer’s instructions [12]. 

### 3.10. Statistical Data Analysis

Data were analyzed with SPSS software, Version 11.5 from SPSS Inc. (Chicago, IL, USA). For multiple comparisons between groups, ANOVA with Bonferroni post-hoc test was used. Results are expressed as the mean ± standard deviation (SD) of the mean. The values with *p* < 0.05 were statistically significant and depicted by a single asterisk in the figures. 

## 4. Conclusions

We investigated the immunity-promoting activity of pRCPS in Cy-immunosuppressed mice. Our findings indicated that pRCPS significantly improved the humoral and cellular immune function of immunosuppressed mice and suggested that the phosphorylated modification potentiated the immunoenhancing activity of RCPS. Thus, pRCPS could be considered as a potential new immune-enhancing drug. However, the detailed mechanisms to elucidate the phosphorylated modification that affects the activity of RCPS needs to be investigated further.

## Figures and Tables

**Table 1:** Treatment of animal groups.

Group	Cy or Saline	Compounds
Normal control	Physiological saline (0.9%)	Physiological saline (0.9%)
Cy	Cy (80 mg/kg/d)	Physiological saline (0.9%)
RCPS+Cy	Cy (80 mg/kg/d)	150 mg/kg B.W. RCPS
pRCPS 50+Cy	Cy (80 mg/kg/d)	50 mg/kg B.W. pRCPS
pRCPS 150+Cy	Cy (80 mg/kg/d)	150 mg/kg B.W. pRCPS
pRCPS 250+Cy	Cy (80 mg/kg/d)	250 mg/kg B.W. pRCPS
Positive control	Cy (80 mg/kg/d)	10 mg/kg B.W. Levamisole hydrochloride

## Abbreviations

RCPS	*Radix Cyathulae officinalis Kuan* polysaccharides
FACS	fluorescence activated cell sorting
FITC	Fluorescein isothiocyanate
IL-2	interleukin-2
IL-4	interleukin-4
IL-5	interleukin-5
IL-6	interleukin-6
IL-10	interleukin-2
IFN-γ	interferon-γ
LPS	lipopolysaccharide
ConA	Concanavalin A
IR	infrared
PM	peritoneal macrophages
